# Comparison of Blood RNA Extraction Methods Used for Gene Expression Profiling in Amyotrophic Lateral Sclerosis

**DOI:** 10.1371/journal.pone.0087508

**Published:** 2014-01-27

**Authors:** Nadhim Bayatti, Johnathan Cooper-Knock, Joanna J. Bury, Matthew Wyles, Paul R. Heath, Janine Kirby, Pamela J. Shaw

**Affiliations:** Sheffield Institute for Translational Neuroscience, Department of Neuroscience, University of Sheffield, Sheffield, United Kingdom; Louisiana State University Health Sciences Center, United States of America

## Abstract

Amyotrophic lateral sclerosis (ALS) is a neurodegenerative disease that causes death within a mean of 2–3 years from symptom onset. There is no diagnostic test and the delay from symptom onset to diagnosis averages 12 months. The identification of prognostic and diagnostic biomarkers in ALS would facilitate earlier diagnosis and faster monitoring of treatments. Gene expression profiling (GEP) can help to identify these markers as well as therapeutic targets in neurological diseases. One source of genetic material for GEP in ALS is peripheral blood, which is routinely accessed from patients. However, a high proportion of globin mRNA in blood can mask important genetic information. A number of methods allow safe collection, storage and transport of blood as well as RNA stabilisation, including the PAXGENE and TEMPUS systems for the collection of whole blood and LEUKOLOCK which enriches for the leukocyte population. Here we compared these three systems and assess their suitability for GEP in ALS. We collected blood from 8 sporadic ALS patients and 7 controls. PAXGENE and TEMPUS RNA extracted samples additionally underwent globin depletion using GlobinClear. RNA was amplified and hybridised onto Affymetrix U133 Plus 2.0 arrays. Lists of genes differentially regulated in ALS patients and controls were created for each method using the R package PUMA, and RT-PCR validation was carried out on selected genes. TEMPUS/GlobinClear, and LEUKOLOCK produced high quality RNA with sufficient yield, and consistent array expression profiles. PAXGENE/GlobinClear yield and quality were lower. Globin depletion for PAXGENE and TEMPUS uncovered the presence of over 60% more transcripts than when samples were not depleted. TEMPUS/GlobinClear and LEUKOLOCK gene lists respectively contained 3619 and 3047 genes differentially expressed between patients and controls. Real-time PCR validation revealed similar reliability between these two methods and gene ontology analyses revealed similar pathways differentially regulated in disease compared to controls.

## Introduction

Amyotrophic lateral sclerosis (ALS) is a devastating and fatal disease that preferentially affects the motor system. Clinically, ALS manifests as progressive weakness of voluntary muscles and patients survive on average for 2–3 years after onset of symptoms. The mechanisms that cause neurodegeneration in ALS are incompletely understood, and are considered to operate through a number of molecular and genetic pathways including glutamate toxicity, oxidative stress, the formation of protein aggregates and defects in axonal transport [1], [2]. Therefore the identification of biomarkers that may detect the early signs of ALS, assess disease progression, monitor the effects of treatment, or even help identify the cause of the disease is of great importance.

Gene expression profiling (GEP) is a powerful tool to help identify potential diagnostic and therapeutic targets in neurological diseases [3]–[5]. Analysis of global expression patterns and differentially expressed genes in an unbiased manner allows for identification of affected functional categories or specific pathways. One potential source for genetic material in this type of study is peripheral blood, which is routinely and easily accessed from patients [3]. Although GEP of whole blood is informative in studying the mechanisms and pathogenesis of a number of diseases, including neurological disorders, the high proportion of globin mRNA present in red blood cells masks potentially important genetic information, and increases noise, thereby reducing sensitivity [6], [7].

Conventional methods for reducing the relative amounts of globin mRNA in blood samples by density gradient centrifugation and extraction of the white blood cell population have been shown to be effective in reducing globin interference. However, the long duration of experimental handling during the extraction process leads to RNA degradation and possible unintended gene induction that might affect the validity of the disease related changes in gene expression [8]. Methods that allow rapid RNA stabilization, and long-term storage without degradation are commercially available e.g. PAXGENE (Qiagen) and TEMPUS (Applied Biosystems) and are currently being utilised (e.g., [6], [9]–[11]). These methods extract RNA from whole blood and so will be affected by globin interference, unless an additional globin depletion step is completed. An alternative strategy has been developed that aims to specifically reduce the amount of globin contamination by enriching the leukocyte population through immobilisation on a filter (LEUKOLOCK, Ambion), thereby avoiding a globin depletion step [12], [13].

In order to discover the best method of RNA isolation from blood to carry out GEP in a neurological disorder such as ALS, we compared these three commercially available systems for RNA extraction from blood. PAXGENE (PAX) and TEMPUS (TEM), which are column based methods leading to RNA extracted from whole blood, and LEUKOLOCK (LL), a method for isolating white blood cells for subsequent RNA extraction. We used a cohort of 8 sporadic ALS patients and 7, age-matched controls. RNA from PAX and TEM extractions also underwent a step to deplete globin using GlobinClear (GC, Ambion). Therefore, RNA from 5 experimental conditions (TEM, TEM+GC, PAX, PAX+GC, and LL) was hybridized onto U133 Plus 2.0 Human whole genome arrays (Affymetrix) and analysed. A number of genes found to be dysregulated in disease compared to controls were confirmed by real-time PCR. This allowed us to identify which method/condition is best suited for carrying out GEP in ALS.

## Results

### RNA extraction, quality and quantity

**Figure 1:**
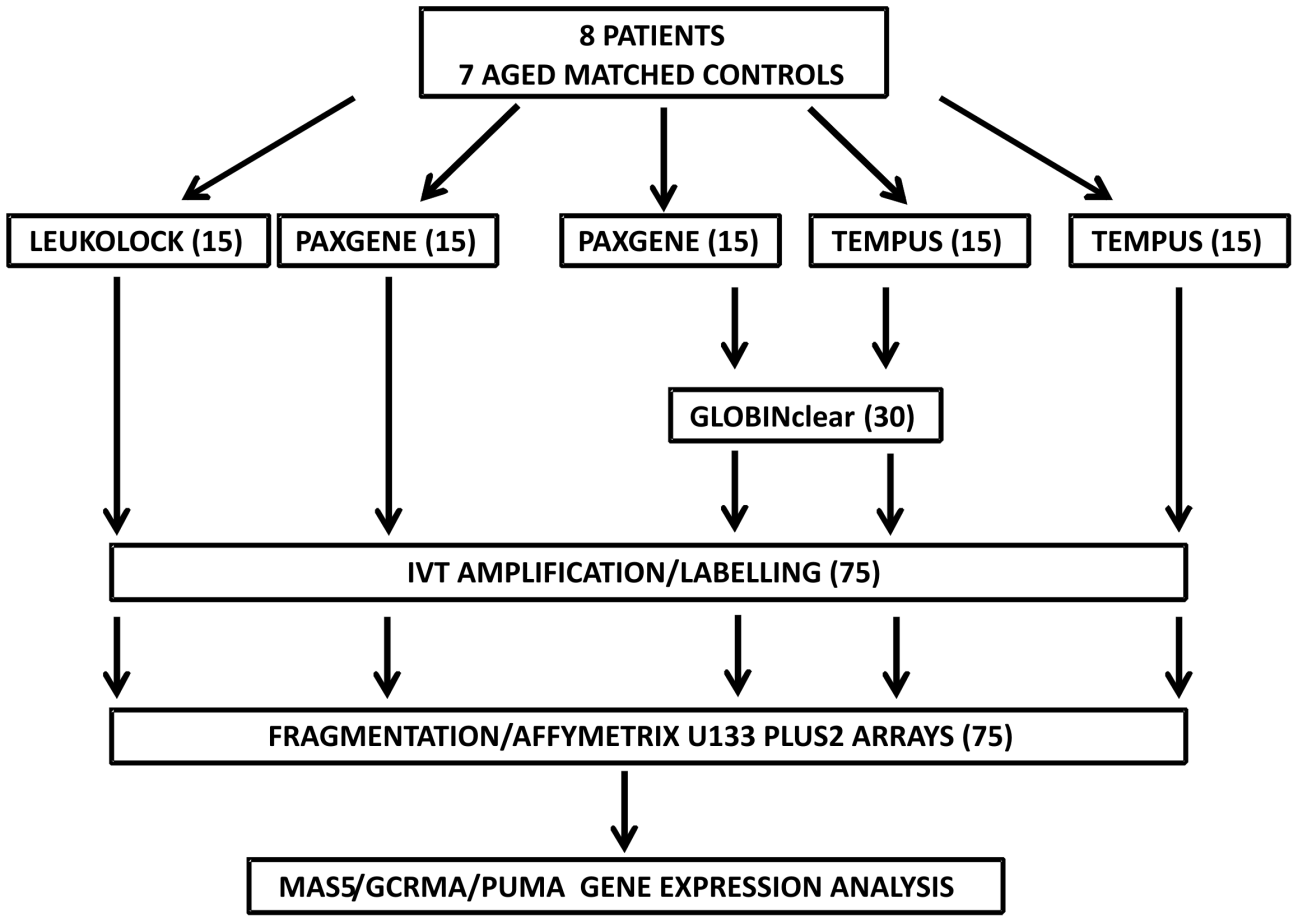
Experimental Design. 5 blood samples were drawn from each of 8 patients and 7 controls were extracted by three methods: LEUKOLOCK, LL; PAXGENE, PAX(x2); TEMPUS, TEM(x2). Samples were extracted separately and not pooled. Additional aliquots of RNA extracted from PAX and TEM samples were depleted for globin RNA using GlobinClear. The resulting 75 samples were amplified by IVT and run on Affymetrix U133 Plus 2.0 whole human genome arrays and analysed with MAS5.0, GCRMA or with the R package PUMA.

**Figure 2:**
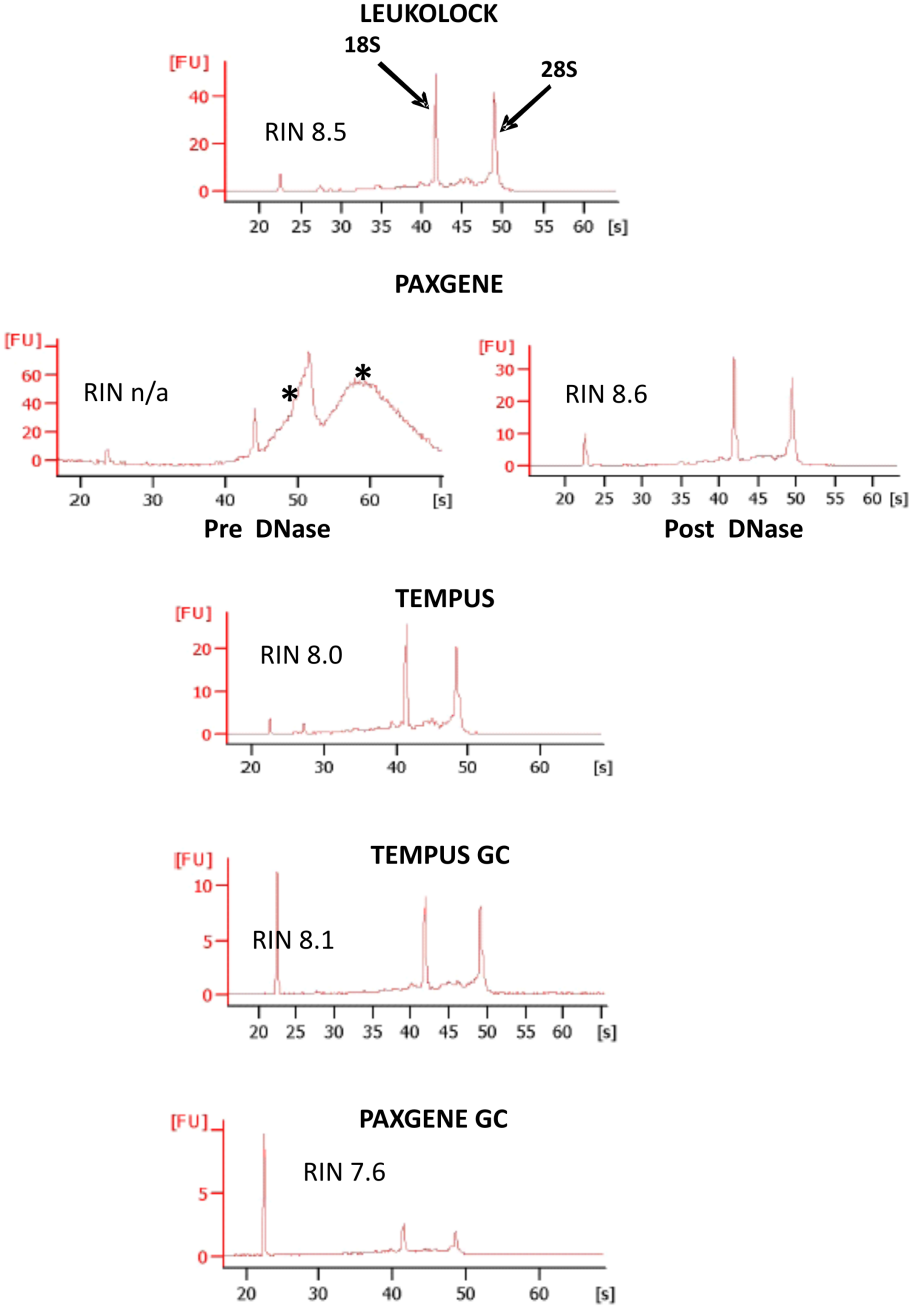
Representative Bioanalyzer electropherograms after initial RNA extractions. High quality RNA (RIN >7.0) can be extracted from all three methods. PAXGENE however requires a DNase step as traces from initial extractions show genomic DNA contamination at high molecular weights and as a “shoulder” to the 28S peak (indicated by asterisks). Good quality RNA can be detected after globin depletion with both TEM and PAXGENE. However, PAXGENE samples showed consistently lower concentration levels, as indicated by the smaller 18S and 28S peaks (FU, fluorescent units). Traces are representative and from single samples from each extraction method.

RNA was extracted by PAX, TEM and LL as described in the methods (**Figure 1**). TEM and LL both had optional DNase steps which were omitted, and the on-column DNase step in PAX was also not carried out to avoid potential damage to RNA quality and yield. The quality and yield of extracted RNA was analysed by examining electropherogram traces generated by the Agilent Bioanlayzer running the samples on a total eukaryote RNA nano chip, and by UV spectrography using Nanodrop. Representative Bioanalyzer traces show that in the cases of TEM and LL, high quality RNA (RIN >7.0) can routinely be extracted (**Figure 2**). However, in the case of PAX extracted RNA, genomic DNA contamination led to a skewing of the 28S peak, and occasionally an additional genomic DNA peak was observed. Therefore all PAX samples were subjected to DNase treatment. No such contamination was observed with TEM and LL. After DNase digestion, high quality RNA was extracted with no signs of genomic DNA contamination, however this resulted in decreased RNA yield. Globin mRNA depletion (GC) using the Globin Clear kit was carried out using 1 µg of total RNA as input for each sample.

**Figure 3:**
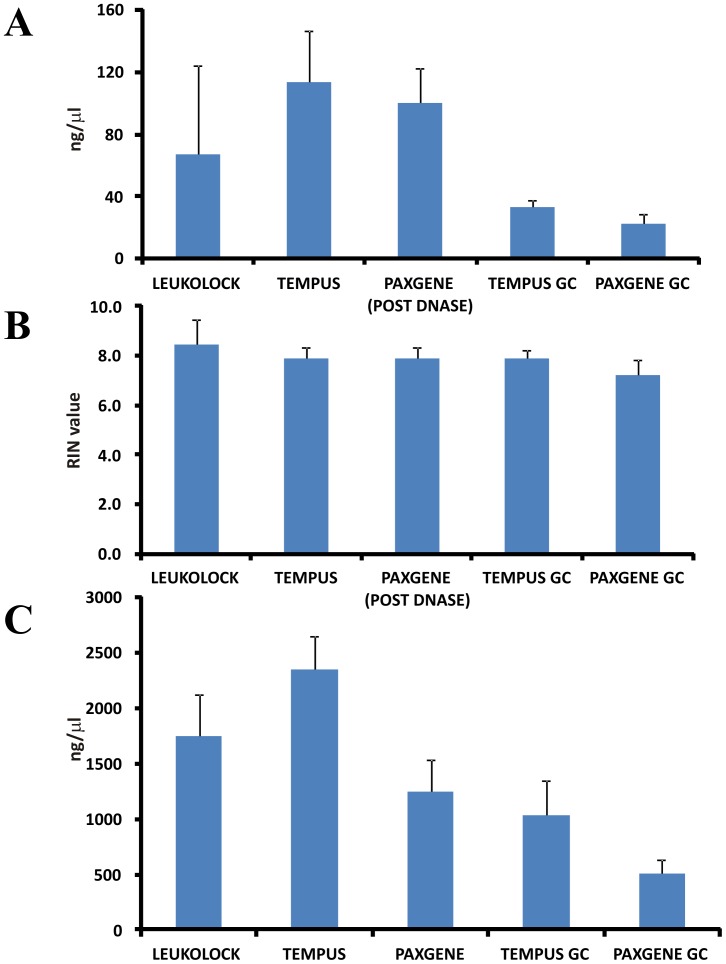
RNA quality and quantity, pre and post in vitro transcription. Diagrams showing averages of all samples in the extent of yield from the 5 experimental conditions (with standard deviation) for (A) RNA extracted, and (C) aRNA produced post IVT, as well as (B) average RIN values of extracted RNA.

Averages of RNA yield and RNA quality (RIN values) for each method were quantified using the Nanodrop and Bioanalyser and are displayed in **Figures 3A and B**. In terms of yield, quantity of RNA was as follows: TEM > PAX > LL and TEM GC > PAX GC (**Figure 3A**). RNA quality as measured by RIN values showed little difference between samples, however LL tended to produce samples with higher average RIN values, while RIN values from PAX GC were the lowest. Although there was a reduction in RIN values after GC in the case of PAX, the RIN values of TEM GC samples remained high (LL, RIN = 8.5, ±1.0; TEM, RIN = 7.9, ±0.44; PAX, RIN = 7.9, ±0.44; TEM GC 7.9, ±0.3; PAX GC 7.2, ±0.59, see **Figure 3B**). PAX required the longest time and most experimental manipulation to carry out, while there were significantly less steps and time required for the other methods.

### aRNA amplification and fragmentation

**Figure 4:**
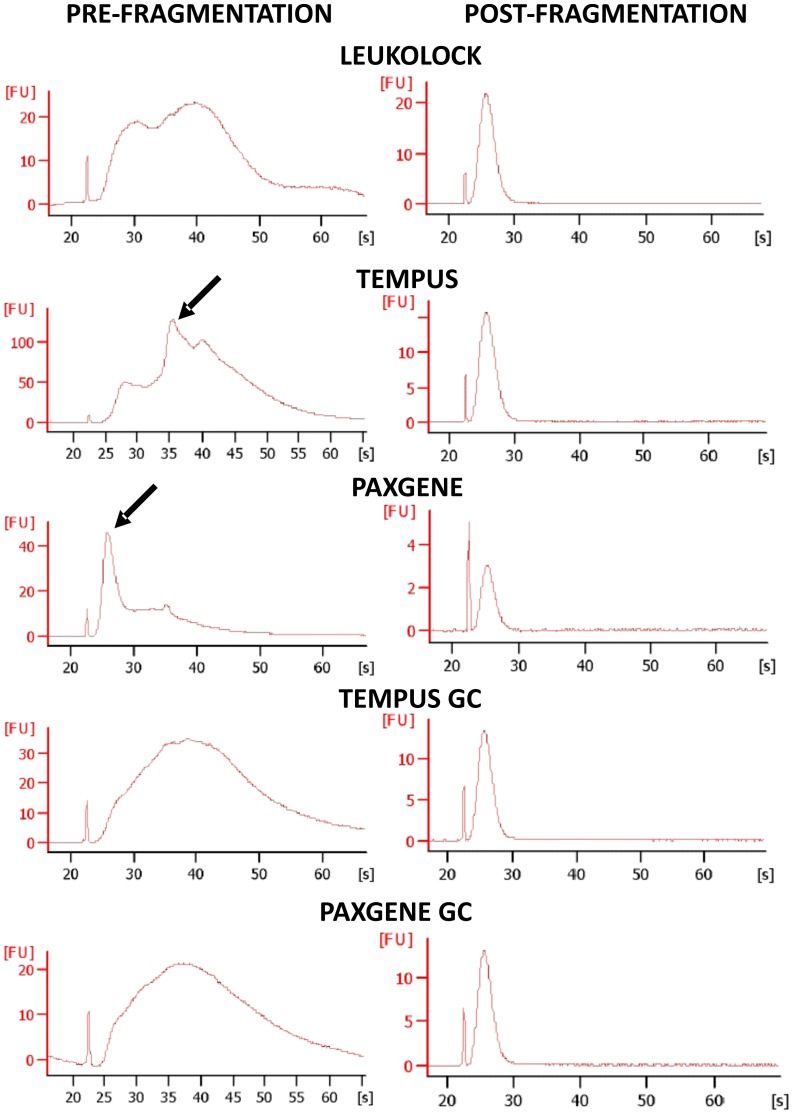
Representative Bioanalyzer electropherograms after IVT and labelling, pre- and post- fragmentation. After IVT, the aRNA profiles from each method showed distinctly different profiles. Generally, IVT resulted in a wide range of detectable products. However in the cases of TEM and PAX especially, distinct high intensity peaks were observed in all cases (black arrows). These disappeared in all cases in the globin mRNA depleted samples. After fragmentation, aRNA profiles were indistinguishable from each other (FU, fluorescent units). Traces are representative and from single samples from each extraction method.

After the *in vitro* transcription (IVT) reaction all samples yielded enough material to progress to the hybridisation step (**Figure 3C**), however some PAX samples required concentrating with Glycoblue. **Figure 4** shows representative Bioanalyzer traces from pre- and post- fragmentation amplified RNA (aRNA). Each method presented a distinctive aRNA profile. However, after fragmentation the profiles were not distinguishable. The profiles of LL, TEM GC, and PAX GC exhibited IVT products over a wide range of molecular weight sizes while PAX and TEM profiles displayed high intensity peaks at low or medium nucleotide sizes, either indicating degradation of product, or the high expression levels of a particular species of RNA that disappeared upon globin mRNA depletion. All samples were hybridised onto Human Genome U133 Plus 2.0 arrays.

### Array hybridisation and Quality Control

**Figure 5:**
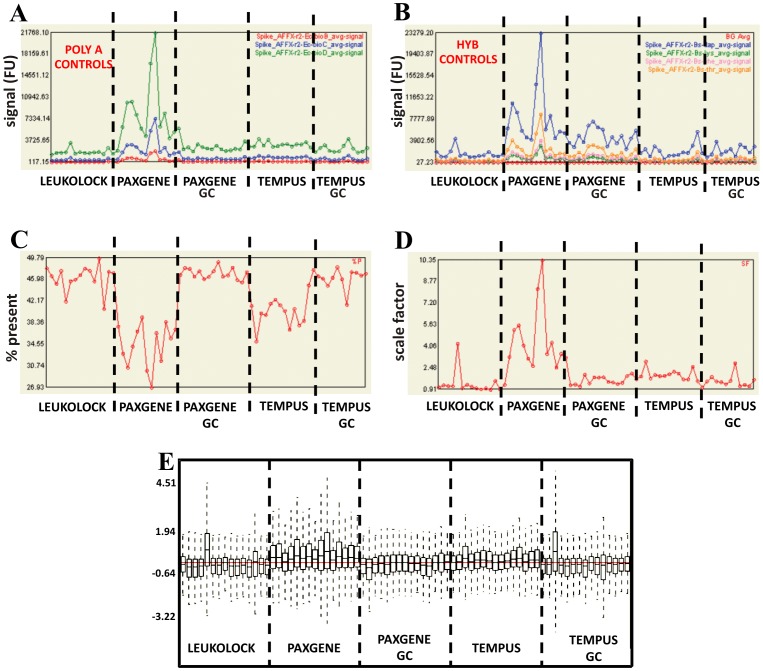
Initial quality control for the 75 microarrays. Analysis of array data of all 75 samples run was carried out in Affymetrix Expression Console. PolyA (A) and Hyb (B) controls show that the preparation steps were consistent, and that in general all hybridisations were consistent, but that hybridisation of PAX samples differed from the rest. The % present call (C) values were generally consistent in LL, TEM GC and PAX GC samples, but were clearly lower with TEM and PAX gene samples. The scale factor between chips were generally low, except for PAX hybridisations (D) while the relative log expression (E) of all chips were generally similar with a few exceptions, and a slight general increase in PAX samples (FU, fluorescent units).

Quality control (QC) for the 75 hybridised arrays was carried out. The polyA controls showed that there was no bias between high and low expressed genes during the preparation steps (**Figure 5A**). However, the different patterns of hybridisation controls (**Figure 5B**) indicate that the conditions of RNA extraction affect PAX hybridisations, causing a highly variable profile which disappears upon globin depletion. The % present calls (**Tables 1** **and** **2** **,** **Figure 5C**) are all consistent (∼40–50%), except for PAX (∼27–39%) and TEM (∼35–43%) which are clearly lower than the others. Scale factors (**Figure 5D** **,** **Table 1** **and** **2**), were all consistent within groups, and comparing between groups except PAX samples which were higher than the rest. Only a few samples showed RNA degradation, and they were included in the further analysis if the scale factor was not affected. The relative log signal of all arrays was generally similar apart for the outliers LL12 and TGC12 (**Figure 5E**), and in general signals from PAX hybridisations showed more variability. These observations indicate that the presence of globin mRNA is an important factor affecting hybridisation quality control. Of the 75 arrays, 72 passed quality control. Three arrays were excluded for not passing QC standards, including scale factor and outlying box plots.

**Table 1:** Selected QC metrics for LL, PAX GC, and TEM GC arrays.

SAMPLE	SF	RawQ	BG Avg	%P	Signal(All)	Actin Signal	Actin 3-5-ratio
**L11.mas5**	5.41	1.069	36.00	48.03	800.34	24743.10	1.70
**L17.mas5**	6.18	0.895	32.19	46.62	834.18	23757.47	1.56
**L9.mas5**	5.91	0.988	33.04	45.26	844.21	25644.59	1.51
**LL10.mas5**	5.80	0.933	32.05	47.53	835.55	27377.06	1.89
**LL13.mas5**	5.12	1.105	37.56	45.75	835.02	25089.33	1.79
**LL14.mas5**	6.41	0.886	32.41	46.01	862.30	25579.43	2.20
**LL15.mas5**	5.58	0.979	34.04	46.83	807.15	22601.53	1.81
**LL16.mas5**	5.10	1.072	36.19	47.97	825.45	25326.25	1.59
**LL3.mas5**	4.69	1.069	36.63	47.58	838.55	25897.72	2.53
**LL4.mas5**	5.04	1.123	36.98	45.68	855.88	27424.78	1.98
**LL5.mas5**	4.55	0.975	33.86	49.79	800.92	21785.49	2.00
**LL6.mas5**	7.74	0.927	31.35	40.82	852.72	11837.65	275.17
**LL7.mas5**	4.77	1.017	34.77	47.43	805.97	23906.62	1.98
**LL8.mas5**	6.29	1.013	36.10	47.17	865.76	26674.71	3.14
**AVERAGE LL**	**5.61**	**1.004**	**34.51**	**46.61**	**833.14**	**24117.55**	**21.49**
**PGC10.mas5**	6.18	0.929	33.08	46.85	837.54	33574.54	1.93
**PGC11.mas5**	6.47	0.786	29.42	48.07	854.88	20879.55	8.82
**PGC12.mas5**	5.69	0.981	34.25	47.93	846.99	30065.17	2.15
**PGC13.mas5**	10.07	0.815	29.56	46.55	853.42	28817.46	3.72
**PGC14.mas5**	6.85	0.867	30.80	47.52	859.75	39770.83	1.98
**PGC15.mas5**	9.06	0.794	30.19	46.09	840.79	36071.60	1.91
**PGC16.mas5**	9.00	0.758	28.10	46.52	839.78	33665.33	2.00
**PGC17.mas5**	9.11	0.809	30.21	47.53	831.49	36207.19	2.09
**PGC3.mas5**	7.46	0.853	31.14	49.12	799.54	22522.94	3.76
**PGC4.mas5**	7.20	0.841	31.68	46.57	834.47	24207.18	2.05
**PGC5.mas5**	6.67	0.805	30.30	46.76	848.39	27207.11	4.63
**PGC6.mas5**	7.05	0.810	30.77	48.14	857.44	31168.63	2.60
**PGC7.mas5**	9.54	0.797	28.21	45.95	824.95	25551.37	3.16
**PGC8.mas5**	10.50	0.837	30.15	45.54	878.26	25963.42	6.24
**PGC9.mas5**	8.41	0.824	30.36	47.32	840.14	31464.83	2.16
**AV. PAX GC**	**7.95**	**0.834**	**30.55**	**47.10**	**843.19**	**29809.14**	**3.28**
**TGC10.mas5**	7.25	0.742	28.55	46.77	849.67	26659.93	3.11
**TGC11.mas5**	7.68	0.850	30.91	44.99	894.94	34257.72	2.38
**TGC13.mas5**	5.35	0.929	32.32	47.71	830.59	23271.15	1.65
**TGC14.mas5**	7.54	0.907	31.90	46.60	887.95	17859.96	4.57
**TGC15.mas5**	9.20	0.877	30.67	46.17	898.22	24888.27	2.78
**TGC16.mas5**	7.55	0.899	31.09	44.94	863.72	28973.61	1.98
**TGC17.mas5**	7.32	0.774	30.01	46.33	854.87	28358.92	2.52
**TGC3.mas5**	6.54	0.914	32.23	48.20	842.43	26744.92	1.67
**TGC4.mas5**	7.79	0.804	29.63	46.08	871.79	22305.48	11.67
**TGC5.mas5**	14.24	0.809	31.02	41.56	1046.02	28440.89	84.00
**TGC6.mas5**	5.84	1.103	36.25	47.36	892.02	30772.99	1.78
**TGC7.mas5**	6.34	0.876	31.12	47.24	830.87	27208.15	1.98
**TGC8.mas5**	5.83	0.866	30.90	46.73	824.06	24356.15	1.61
**TGC9.mas5**	8.18	0.807	29.94	47.05	851.78	23093.71	2.32
**AV. TEM GC**	**7.62**	**0.868**	**31.18**	**46.27**	**874.21**	**26227.99**	**8.86**

Scale factor (SF), rawQ, background (BG), percentage present (%P), total signal, actin signal, as well as actin 3′ to 5′ ratio are all listed for LL, PAX GC and TEM GC arrays used for this study. Three arrays in total did not pass quality control and were not analysed: one array was extracted by the LL method, one array by TEM GC and one from TEM.

**Table 2:** Selected QC metrics for PAX and TEM arrays.

SAMPLE	SF	RawQ	BG Avg	%P	Signal(All)	Actin Signal	Actin 3-5-ratio
**P13.mas5**	23.86	0.832	29.48	33.74	910.39	41134.71	2.37
**P16.mas5**	20.97	0.805	30.11	33.61	908.23	35056.00	2.30
**PG10.mas5**	16.53	0.779	29.05	37.72	917.59	46817.15	2.25
**PG11.mas5**	26.34	0.784	29.39	32.91	975.39	58756.44	3.95
**PG12.mas5**	28.01	0.800	30.19	30.45	949.32	38274.88	3.03
**PG14.mas5**	20.69	0.773	29.40	34.20	937.87	49253.10	2.31
**PG15.mas5**	15.86	0.877	31.80	36.87	873.14	38324.91	2.23
**PG17.mas5**	13.24	0.792	29.55	39.35	848.18	33711.05	2.80
**PG3_1.mas5**	41.31	0.706	27.23	29.97	997.94	49350.14	31.60
**PG4_1.mas5**	51.73	0.744	28.46	26.93	1016.88	31745.37	48.67
**PG5.mas5**	17.60	0.769	28.84	36.57	959.76	45626.00	2.13
**PG6.mas5**	21.69	0.840	30.15	31.64	927.78	42699.89	3.18
**PG7.mas5**	12.57	0.930	32.50	38.57	867.41	33979.42	2.09
**PG8.mas5**	17.66	0.851	30.08	35.65	906.00	35655.77	2.38
**PG9.mas5**	16.20	0.839	31.17	37.20	886.68	24967.63	1.73
**AV. PAX**	**22.95**	**0.808**	**29.83**	**34.36**	**925.51**	**40356.83**	**7.53**
**T10.mas5**	6.83	0.896	32.21	43.27	841.55	25843.87	1.67
**T11.mas5**	9.42	0.883	32.82	41.37	882.21	29647.80	1.42
**T12.mas5**	14.88	1.009	33.58	35.12	942.61	33275.39	1.37
**T13.mas5**	8.67	1.037	35.47	40.05	893.06	29685.47	1.38
**T15.mas5**	13.33	0.873	30.23	37.99	897.58	26182.47	1.60
**T16.mas5**	10.01	0.987	32.56	39.78	933.23	28284.56	1.25
**T17.mas5**	9.47	0.864	31.85	41.78	877.22	28296.21	1.23
**T3_1.mas5**	9.61	0.990	32.90	42.47	893.88	32139.52	1.46
**T4.mas5**	10.17	0.959	33.49	41.51	886.43	28244.28	1.44
**T5.mas5**	11.12	0.953	32.65	40.48	872.70	24144.52	1.66
**T6.mas5**	10.41	1.127	37.35	37.16	884.89	33334.57	1.30
**T7.mas5**	8.29	1.138	38.70	40.82	909.65	38670.56	1.40
**T8.mas5**	8.37	1.144	37.47	37.94	914.99	40451.85	1.26
**T9.mas5**	12.92	0.887	31.10	38.73	893.45	28633.24	1.56
**AV. TEM**	**10.25**	**0.982**	**33.74**	**39.89**	**894.53**	**30488.16**	**1.43**

Scale factor (SF), rawQ, background (BG), percentage present (%P), total signal, actin signal, as well as actin 3′ to 5′ ratio are all listed for PAX and TEM arrays used in this study. Three arrays in total did not pass quality control and were not analysed: one array was extracted by the LL method, one array by TEM GC and one from TEM.

**Figure 6:**
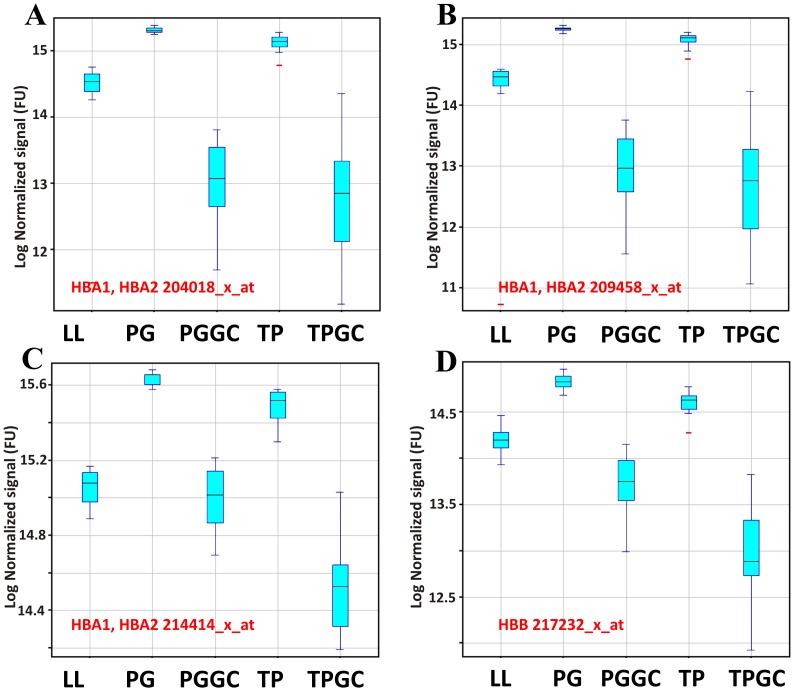
Quality control: globin levels in arrays. Data were GCRMA normalised and levels of 3 representative alpha globin probe sets (A, B and C) and one beta probe set (D) were averaged for each condition. Expression level box plots indicate that in both the cases of alpha and beta globin, TEM and PAX exhibit highest levels of globin mRNA while TEM GC and PAX GC were the lowest, LL levels were generally lower than PAX and TEM, but higher than PAX GC and TEM GC.

Further quality control was carried out on GCRMA normalised data to check the effects of GC on globin RNA levels. Genespring 12.5 (Agilent) was used to plot the average expression levels of a number of probe sets in the highest expressing human globin isoforms (alpha and beta) for each of the five conditions. In the case of alpha globin probe sets, representative plots (**Figure 6A, B, C**) indicate that alpha globin levels are highest in PAX and TEM samples, while LL are lower, and TEM GC and PAX GC exhibit the lowest levels of alpha globin mRNA levels. A similar pattern is also seen in the case of beta globin (**Figure 6D**).

**Figure 7:**
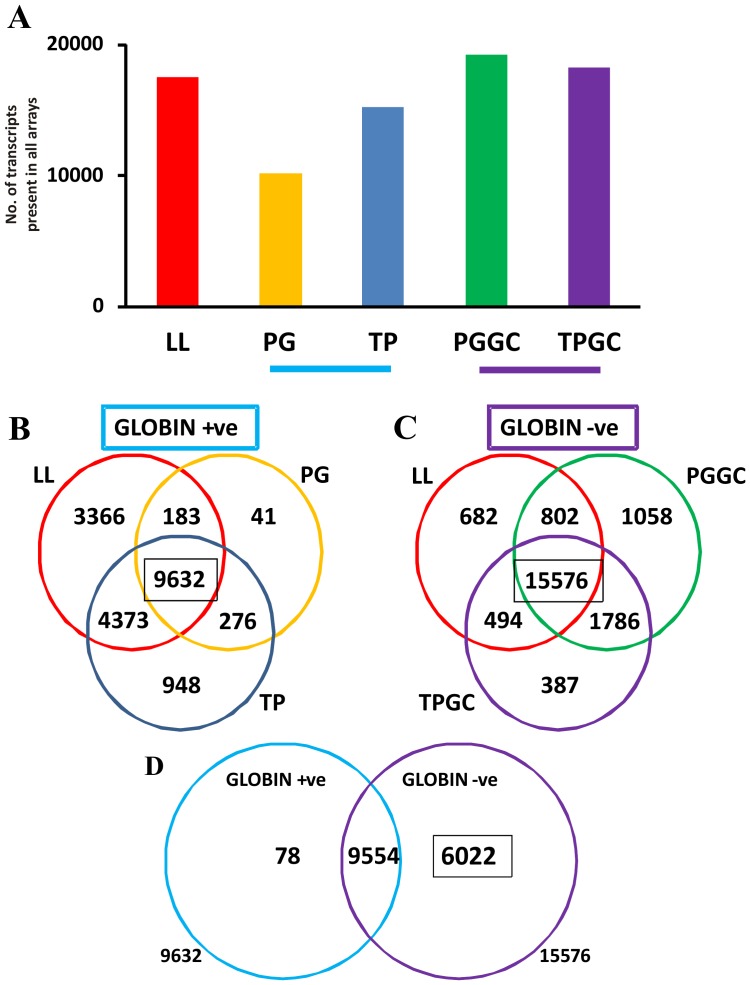
Number of probe sets called present increases after globin depletion. MAS5.0 normalised data indicated that the presence of globin affects the number of probes sets called present. Comparison of all probe sets called present in every array for each condition reveals higher numbers in the case of LL, TEM GC and PAX GC (“globin negative”) as compared to TEM and PAX (“globin positive”; A). Comparison between probe sets called present in LL, TEM and PAX reveals 9632 probe sets in common (B), and 15576 probe sets were found to be in common between LL, TEM GC and PAX GC (C). By comparing these 2 populations 6022 probe sets were found to be unmasked by globin depletion (D).

Analysis in MAS5.0 was carried out to determine the effect of globin clearance on transcripts present. Analysis of the number of transcripts present in all arrays for each condition (**Figure 7**) indicated that globin clearance unmasks a large percentage of transcripts that would otherwise not be detected. Comparing all arrays in each condition for transcripts called present (**Figure 7A**), indicated that arrays from “globin positive” conditions (PAX and TEM) exhibited far less present transcripts than “globin negative”, globin depleted samples from PAX GC and TEM GC. The LL method which enriches RNA from leukocytes revealed present calls similar to the “globin negative” group of conditions. This pattern was also similar when comparing present calls in either controls only or patients only (data not shown). Venn diagrams comparing number of present calls between LL, PAX and TEM showed 9632 probe sets in common, while a comparison between LL PAX GC and TEM GC, exhibited 15576 probe sets in common (**Figure 7B, C**). A further comparison between the these two populations of transcripts indicated 6022 probe sets in the population of 15576 that were called present in the “globin negative” group representing genes unmasked by globin depletion (**Figure 7D**). DAVID analysis was carried out with the 6022 probe sets. Genes associated with intracellular organelles, most notably the nucleus and mitochondria were identified, including many related to transcription (see **Table S1**).

### Gene expression analysis

**Figure 8:**
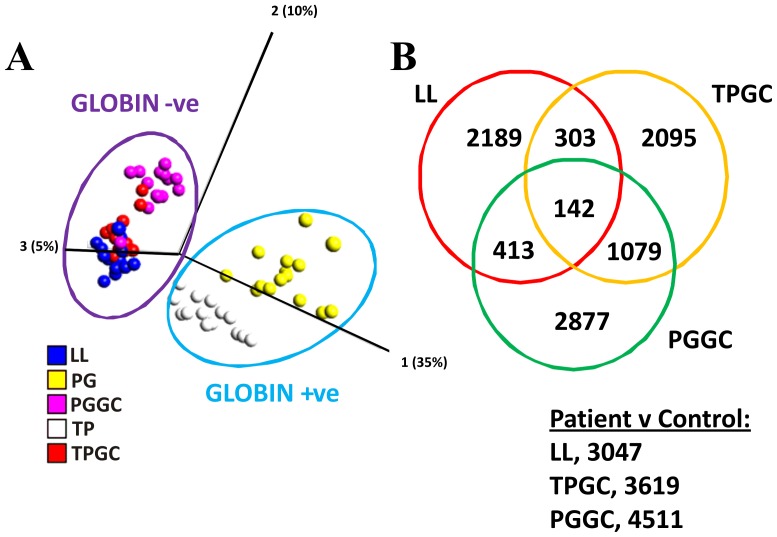
Gene expression analysis. PCA plotted using QLUCORE OMICS Explorer on GCRMA normalised data: each dot represents an array and takes into account the expression levels of every probe set and shows 2 main clusters (A). One tightly clustering ‘globin negative’ cluster includes the following conditions: LL, TEM GC and PAX GC, while a second more variable cluster “globin positive” includes arrays hybridised with PAX and TEM extracted RNA. Focusing on the globin negative group PUMA analysis identified differentially regulated probe sets (ALS patients vs controls) for the following (B; LL, 3047; TEM GC, 3619; PAX GC, 4511). A Venn diagram comparing these lists shows that TEM and PAX share more genes in common, than either with LL, and 142 genes in common between all 3 methods).

Using QLUCORE OMICS Explorer, principal component analysis (PCA) on GCRMA normalised samples that took into account the expression levels of all probe sets on the arrays showed that 2 main clusters were visible and broadly corresponding to globin levels (**Figure 8A**). A tightly related “globin negative” cluster included the following conditions (LL, TEM GC and PAX GC), while a more variable “globin positive” cluster (PAX and TEM) was also present. Taking together this observation with the potential importance of 6022 probe sets uncovered by globin depletion to the ontology of ALS, subsequent gene expression analysis was carried out on the conditions in the “globin negative” group while “globin positive” samples were excluded.

A PUMA analysis of differentially-regulated genes between patients and controls was carried out, and the three conditions revealed the following number of statistically significant (p≤0.05) differentially regulated probe sets: LL, 3047; TEM GC, 3619; PAX GC 4511 (**Figure 8B**). A Venn diagram comparing these results showed that 142 probe sets were in common between the three conditions, while TEM GC and PAX GC shared more common probe sets than either LL and TEM GC or LL and PAX GC. Due to the longer preparation time and additional steps required for PAX GC, PCR validation of gene lists were only carried out in the case of LL and TEM GC conditions (see **Tables S2** and **S3** for identities of differentially regulated probe sets for LL and TEM GC respectively). A control analysis was also carried out in order to measure the probability of measuring the number of differentially regulated genes in any two random groups of people of a similar size. This would help identify the amount of noise in our methods. The 15 individuals were randomly sorted into two groups five times (not based on ALS status) and these groups were compared against each other. PUMA analysis carried out for each random group pairing demonstrated the number of differentially regulated genes between groups was lowest in the case of LL with an average of 37.74% ±10.54 of the original number of genes. TEM GC exhibited more noise, with an average of 46.91% ±18.49 decrease compared to the number of genes in the original study (**Table S4**).

### Real time PCR validation

**Figure 9:**
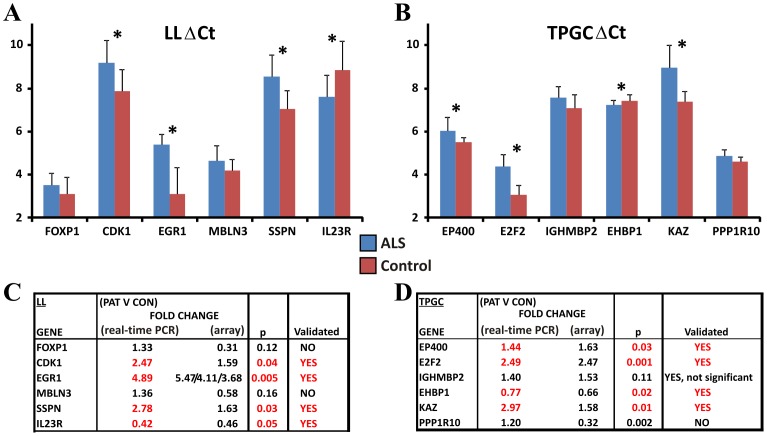
Real-time validation PCR for genes in LL and TEM GC gene lists. ΔCTs plotted for 6 genes chosen for validation using the LL method of RNA extraction from blood (A). Four out of 6 genes (CDK1, EGR1, SSPN and IL23) could be validated from the Affymetrix microarray studies (C). ΔCTs plotted for 6 genes chosen for validation using the TEMPUS/GC method of RNA extraction from blood (B). Four out of 6 genes (EP400, E2F2, EHBP1 and KAZ) could be validated from the Affymetrix microarray studies (D). Blue bars represent levels of gene expression in ALS patients (n = 6) and red bars the levels in normal control individuals (n = 6).

To determine the reproducibility of the methods, 6 differentially regulated genes were chosen from each of the LL and TEM GC gene lists for real time PCR validation. These genes exhibited a fold change of at least ±1.5 (patient versus control), with two genes from high, medium and low probability (as calculated by PUMA) values were chosen. PCR calculated fold change values were calculated from patient and control ΔCt values (**Figure 9**).

In the case of LL, 4 out of 6 genes were validated with significance: CDK1, EGR1, SSPN, and IL23R (all p<0.05 with t-test), while 2 genes were not validated as being regulated in the same direction as the arrays: MBLN3 and FOXP1.

In the case of TEMPUS/GC, 4 out 6 genes were validated with significance: EP400, E2F2, EHBP1 and KAZ (all p<0.05), while one gene was regulated in a similar way to that seen in the array without significance: IGHMBP2 (p = 0.11), and 1 gene, PPP1R10, was not found to be regulated in a similar way observed with the arrays.

In summary both methods were able to validate two-thirds (4/6) of the genes chosen thereby indicating that these methods are similarly reliable. However the reliability of a method in terms of gene validation by PCR does not necessarily correlate with the relevance of the method to uncovering genes important in the disease process, therefore gene ontology studies were carried out on the lists of differentially expressed genes identified by the two methods.

### Gene Ontology and KEGG pathway analysis

Initially, functional annotation clustering was carried out using DAVID. In the case of TEMPUS/GC 439 clusters were identified of which the top 15 are ranked (**Table 3**). In the case of LL, when high stringency was used, 383 gene clusters were identified of which the top 15 are ranked in **Table 4**.

**Table 3:** Functional annotation clustering carried out in DAVID for differentially regulated genes from a cohort of ALS patients and controls, using RNA extracted from blood with LL and TEM GC undergoing PUMA GEP analysis.

LEUKOLOCK Annotation Cluster	Enrichment Score	Terms
***1***	***10.86***	***intracellular organelle lumen incuding nucleus***
***2***	***9.29***	***RNA recognition motif/RNP-1***
***3***	***7.41***	***nucleotide binding***
4	7.06	phosphate/phosphorus metabolic processes/phosphorylation
***5***	***6.66***	***transcription/transcription regulation***
6	5.78	zinc finger/zinc/zinc ion binding
7	4.62	serine/threonine protein kinase/protein kinase activity
8	4.23	protein catabolic processes
***9***	***4.21***	***apoptosis/cell death***
10	3.99	helicase activity
***11***	***3.77***	***protein transport/localisation***
12	3.62	Lissencephaly type-1-like homology motif
13	3.21	zinc finger, PHD type
14	3.08	Bromodomains
15	2.83	pleckstrin homology

TEMPUS GC Annotation Cluster	Enrichment Score	Terms
1 and 2	34.43/29.37	Ribosome
***3***	***22.94***	***intracellular organelle lumen incuding nucleus***
4	12.06	RNA splicing
***5***	***6.53***	***regulation of apoptosis***
6	6.51	ribosome biogenesis, RNA processing and metabolic processes
***7***	***6.31***	***RNA recognition motif/binding***
***8 and 9***	***5.58/4.80***	***apoptosis and cell death***
***10***	***4.72***	***protein transport/localization***
11	4.25	mitochondrial membrane
***12***	***4.23***	***nucleotide binding***
13	3.7	lysososome/lytic vacuole
***14***	***3.44***	***transcription/transcription regulation***
15	3.41	Like-Sm ribonucleoprotein (snRNP's)

In the case of LL, out of 383 clusters, the top 15 are ranked in order of enrichment score (upper section). In the case of TEM GC, out of 439 clusters, the top 15 are ranked in order of enrichment score (lower section). Common clusters between LL and TEM GC are labelled in italic and underlined.

**Table 4:** PCR primer sequences designed for genes validated in this study.

LEUKOLOCK		
Gene	Sequence	Product Size
FOXP1 S	ctctgtcatcacaaccaccag	138
AS	ggtggtctaacttctgcgttc	
IL23R S	ggcaagaagtacttggtttgg	128
AS	atagtctcagccctggaaatg	
CDK1 S	aggtcaagtggtagccatgaa	108
AS	tttggatgacgaagttccttt	
EGR1 S	gagaaggtgctggtggagac	142
AS	cactgaccaagctgaagagg	
MBNL3 S	tccatcaattccagctaatcc	112
AS	tccaggaatcagaacaggtg	
SSPN S	ttacctgtgagaccacactcg	108
AS	cagtgacgctggtacagtcc	

TEMPUS GC		
Gene	Sequence	Product Size
PPP1R10 S	acaagccactcaacacaacac	107
AS	ggctgaattaagagcatccag	
KAZ S	ctagccagagccaaagaagc	150
AS	atagttgcggatgaagtctcg	
EHBP1 S	attctgcaacaggcagctaag	109
AS	tgacatcttcactccagatcg	
E2F2 S	gccaagaacaacatccagtg	102
AS	gttcatcagctccttcagctc	
IGHMBP2 S	gctgacattcttcaacacctg	132
AS	atgatctcaaccacagtcgtg	
EP400 S	ctggtcattgatgagatgcag	131
AS	cagagctccaggaaggtattg	

Samples of cDNA were diluted 1∶10, and 2.5 ul were used per PCR reaction for all genes except EHBP1, where 4 µl was used with 5x Brilliant III Ultra Fast SYBR Green QPCR Master Mix (Agilent). In all cases primer concentrations were optimised to 300 mM (S, sense strand; AS, antisense strand)

Although there are similarities between the 2 lists in terms of cluster identification, e.g. nucleus/intracellular organelle, apoptosis/cell death, nucleotide binding, RNA recognition motifs and transcription/transcriptional regulation all being found to be enriched with both methods, there are major differences too. The extent of enrichment in TEM GC for the top 3 clusters: ribosome, nucleus and RNA splicing was much greater than the top 3 clusters for LL (nucleus, RNA recognition motif and nucleotide binding) and of the top 3 clusters in TEM GC, ribosome and RNA splicing were not present in the top 15 LL clusters. (In LL, splicing was ranked as 27, with an enrichment of 2.32, while ribosome biogenesis/RNA processing and metabolic processes was ranked as 267) Of the top 3 LL clusters, all 3 are found with the top 15 TEMPUS/GC clusters.

KEGG pathway analysis was also carried out in DAVID, in order to give an indication of the extent of detected changes in intracellular pathways with each experimental method (See **Tables S5** and **S6**). KEGG analysis of the LL gene list revealed dysregulation of 45 pathways including a number of signalling cascades such as MAPK, Jak-STAT, and phosphatidylinositol and associated receptor-mediated pathways (**Table S5**). KEGG analysis of the TEM GC gene list indicated 33 affected pathways (**Table S6**). Specific signalling cascades observed in the LL list were absent, however a number of common receptor-associated pathways were affected, e.g. Insulin signalling pathway, Neurotrophin signalling, B-cell receptor signalling. Comparing between the two KEGG pathways analyses, LL and TEMPUS share 17 common dysregulated pathways (accounting for 38% of LL pathways, and 52% in the case of TEM GC).

Taken together, this analysis suggests that TEM GC and LL methodologies are similarly consistent when choosing genes to validate by qPCR, and that whilst they do result in similar enrichment in affected gene ontologies, the data from TEMPUS/GC shows more statistical significance.

## Discussion

This study compares three RNA extraction methods from blood and their suitability for GEP in a neurological disease such as ALS. All three methods allow blood drawn to be stored in stabilizing agents and therefore provide alternatives to traditional PMBC isolation which requires the immediate processing of blood samples [14]. Two column based methods (PAX, TEM) resulting in total blood RNA extraction and one method which enriches for the leukocyte population were used (LL). Previous studies indicate that long term storage of blood in RNA stabilizing solutions does not adversely affect the transcript profile in extracted RNA [8], [15]–[17]. However, there have been reported differences between PAX and TEM methods [11], [17], [18]. Here we are the first to compare whole blood extraction methods with a leukocyte enrichment method in human blood. In addition, globin depletion was carried out on PAX and TEM blood samples in order to compensate for the masking effects of high levels of alpha and beta globin transcripts using the commercially available GC method [7], [19]. In total 5 conditions were therefore assessed and compared, LL, TEM, TEM/GC, PAX and PAX/GC.

### Blood extraction and RNA isolation

Blood was drawn directly into TEM and PAX tubes containing RNA stabilizing solutions, the two methods required relatively small amounts of blood (3 ml and 2.5 ml respectively) and stored at −20°C after the required mixing (PAX also requires 2 hours incubation at room temperature before freezing). In the case of LL, a larger quantity of blood, 8–9 ml, was first collected in a standard vacutainer blood collection tube before being filtered into another collection tube. The LL filter required flushing with PBS and saturating with RNAlater before being stored at −20°C directly. For RNA extraction, using the 2 column-based methods, TEM required less time to carry out compared to PAX, also TEM extraction resulted in high yield, high quality pure RNA without the requirement of DNAse step, which was required for PAX, (though this may be accounted for by the slightly smaller amount of blood drawn). LL extraction resulted in intermediate yield but also required less time to carry out than PAX. These observations are in broad agreement with previous studies [17]. Isolation of RNA with the LL method also took less time and experimental manipulation than PAX, and also resulted in high yield, high quality RNA without the need for a DNase step.

### Globin depletion

Globin depletion was carried on PAX and TEM extracted RNA samples using GlobinClear. Levels of the most abundant globin alpha and beta globin isoforms in adult human blood showed reduction in these samples, compared to non-globin cleared samples and averaging lower than levels in LL, but with greater variability. LL samples did exhibit residual levels of globin mRNA, which were also lower than non-globin cleared samples. Previous studies suggested that GC depletion results in lower RNA quality and RIN values [9]. We did not observe any reduction in RNA quality, although the RNA yield was reduced, which was more marked in the PAX samples. GC utilises oligonucleotides that target the 2 most abundant human globin species (alpha and beta, [20]), yet similar reductions in lower expressing isoforms (i.e. delta, epsilon and zeta) were also observed in PAX and TEM GC RNA extractions (data not shown). Globin depletion added an additional step to the extraction protocol and the additional time and cost of the procedure must be taken into account when deciding a suitable method for RNA extraction from blood.

### IVT and fragmentation

All methods/conditions yielded sufficient quality and quantity of RNA to proceed with a reverse transcription reaction. After second strand synthesis, amplification and production of aRNA by *in vitro* transcription was carried out. IVT yield was greatest in the following order: TEM > LL > PAX > TEM GC > PAX GC. Additionally, while PAX yielded on average more RNA after extraction than LL, the latter method resulted in higher levels of aRNA even though the same amount of input cDNA was used in the IVT reaction. Possible reasons for this may be explained by comparing pre-fragmentation profiles of aRNA observed with the different conditions. Intense discrete peaks at low mass nucleotide ranges could be observed in all TEM and PAX aRNA profiles after Bioanalyzer analysis. All TEM profiles exhibited similar peaks, and all PAX profiles exhibited similar peaks. These probably represent aRNA populations associated with globin since following globin depletion these peaks disappeared in all cases and a wide range of RNA populations were observed in both TEM and PAX. A similar broad range of aRNA populations were visible in pre-fragmented LL samples. After fragmentation, profiles were indistinguishable between methods. These observations highlight the importance of globin depletion/reduction in RNA extracted from blood samples, as aRNA populations from globin skew the relative proportions of other aRNA populations within samples.

### Affymetrix array quality control

Quality control metrics were consistent within each sampling method. However, when comparing between methods the saturating presence of globin significantly affected the percentage of present calls using MAS5 analysis. PAX and TEM present calls were distinctly lower than LL, PAX GC and TEM GC. Thus, already we can distinguish two separate groups of arrays, a “globin positive” group with low % present calls/large scale factor, and a “globin negative” group with higher % present calls/low scale factor. The identity of these 2 groups is highlighted in the PCA plot in **Figure 8**. The arrays within the “globin negative” group are much more tightly related than the arrays in the “globin positive” group which exhibit much more variability. These observations were underpinned by an approximate 60% increase in transcript present calls in the “globin negative” group compared to the “globin positive” group (15576 vs 9632 respectively). GO analysis of the approximately 6000 additional transcripts called present in the globin negative group revealed many genes associated with the nucleus, gene transcription and mitochondria. A number of these genes could be potentially be important in the aetiology/progression of ALS, given the current focus on defects in RNA transcription and processing as well as oxidative stress [2]. Levels of alpha and beta globin isoforms mirrored the expected globin status throughout all conditions, and although LL globin levels were higher than TEM GC and PAX GC, they exhibited less variability between samples. When comparing the number of present calls of LL with other conditions, more transcripts were found to be present and in common with TEM GC/PAX GC as compared to TEM/PAX, indicating that residual globin levels in LL do not obscure the visibility of lower expressed transcripts on arrays, in comparison with the GC depleted samples.

### Real-time PCR validation of differentially regulated genes and gene ontology differences

Using the PUMA bioconductor package [21], lists of differentially regulated genes in ALS as compared to controls were produced from LL, TEM GC and PAX GC samples. Comparison of these gene lists indicated that PAX GC and TEM GC shared a larger number of common differentially regulated genes, compared with LL. This difference can be explained when considering that the starting material in PAX and TEM cases was whole blood, containing additional cell populations, while LL enriches for leukocytes. A control analysis was also carried out with LL and TEM GC samples, where the 15 subjects were randomly assigned into 2 groups irrespective of ALS status and gene expression analysis with PUMA was carried out in order to ascertain the probability of finding differentially regulated genes in any two random groups of subjects of a similar size. After 5 iterations, the average number of differentially regulated genes (expressed as a percentage of the original study) was lower in LL than TEM GC, suggesting less noise in the LL data. An average of almost 40% of the original number of genes (approx 1200) is likely to be seen as differentially expressed in any random study. Although a number of these may be bona-fide targets, this highlights the difficulty involved in identification of actual disease markers using the techniques employed in this study.

Due to the technical limitations of the PAX technique (low yield after additional methodological manipulation), this method was disregarded for PCR validation which was carried out on LL and TEM GC samples, as they represent better methods for GEP profiling. Six genes were chosen with a minimum of 1.5 fold difference between disease and controls for each method and real-time PCR resulted in the validation of 4 out of 6 genes for each condition, indicating that both methods present similar consistency and reliability. These similarities in consistency between the 2 methods may not represent similarities in differential regulation of gene pathways, and therefore GO functional annotation clustering analysis was carried out on the respective gene lists to ascertain putative differences/similarities. Considering the a) relatively few differentially regulated genes in common between the 2 methods, and b) the difference in starting material (whole blood versus leukocyte enriched), GO analysis revealed a striking similarity in the number of common cluster terms, and this was supported by similar observations when carrying out KEGG pathway analysis. It is noteworthy that the top cluster terms, ribosome-associated genes, in TEM GC were not present in LL, and this raises the question as to whether these are associated with pathological changes and/or mirror underlying differences in initial cell populations used as starting material for each extraction method. As there are only small differences between globin levels (depleted and undepleted isoforms) in TEM GC samples when comparing patients and controls (data not shown) we can discount any potential sampling/extraction bias leading to the observed differences in ribosome-associated gene expression. Taking into account the relatively small number of ALS patients and controls in this study, drawing conclusions regarding pathway analysis should be cautious, however examples of pathways involved in ALS include: defects in RNA processing [2], and thus changes in expression of ribosome-associated genes during disease may arise from the aggregation of proteins such as TDP-43 and FUS, and down-stream effects e.g. on stress granule formation arising from dissociating ribosomes [22]. In addition, the most striking differences in LL-KEGG pathway analysis were the enrichment for specific intracellular signalling pathways, such as MAPK, and PI3K, cascades commonly involved in signal transduction from the cell membrane and involved in cell survival in ALS [23], [24] as well as the JAK/STAT pathway, a regulator of neuronal apoptosis [25].

## Conclusions

GEP with blood is a feasible approach for biomarker discovery in neurological disease. Overlap in transcript expression and splicing between blood and neural tissues has been demonstrated [6]. The present study primarily highlights the importance of reducing the high levels of globin present when using blood for GEP profiling in neurological disease [7]. After reduction in globin levels, the expression of ∼30% of additional transcripts are unmasked, many with functions of potential importance in the pathophysiology of ALS. Although reduction in globin was achieved through different methods, remarkably similar results were obtained when comparing dysregulated pathways in ALS. The preferred TEM GC and LL methods produced the highest yield, most consistent array performance, and similar reliability when validating gene targets with real-time PCR. PAX was more a labour-intensive method, also produced good quality RNA but with lower yield and, after subsequent globin depletion, RNA quality was reduced. In future biomarker studies when making a decision regarding which method to use, the extra cost and the time required for the globin depletion step in the case of TEM must be considered against the larger volume of blood required for LL. Although we have not analysed whether any of our validated genes correspond to bone-fide hits for biomarkers, future studies should concentrate on the identification and validation of potential targets that may arise as bona-fide biomarkers for ALS. Single targets or clusters of targets can be validated by PCR in either human samples or those from animal models in a blinded fashion to see whether they are predictive of ALS or in the case of serial sampling, of disease progression.

## Methods

### Blood collection

Ethical approval for the study was granted from South Sheffield Ethics Committee and written informed consent from each subject was obtained before commencing. Blood was collected after an overnight fast from patients (n = 8) or age- matched controls (n = 7, see **Table 5**) either directly into PAX/TEM tubes (approx. 3 ml) or into K_2_EDTA spray-coated collection (BD Vacutainer) tubes (approx. 8–9 ml), for immediate passage through a LL filter. RNA was stabilized in PAX and TEM tubes by slow inversion (PAX) or vigorous shaking (TEM) for 30 and 15 seconds respectively. LL filters were washed with PBS and saturated with RNAlater as described in the manufacturer's instructions. Samples were stored at −20°C until processed further.

**Table 5:** Characteristics of subjects taking part in the study.

PATIENT ID	SAMPLE DATE	AGE	GENDER	DIAGNOSIS/RILUZOLE
3	05/04/2012	57	M	ALS/R
4	12/04/2012	53	F	ALS/R
13	19/04/2012	72	M	ALS/-
7	26/04/2012	64	M	ALS/-
8	26/04/2012	60	M	ALS/-
16	11/05/2012	62	F	ALS/R
9	11/05/2012	63	F	ALS/R
17	12/06/2012	71	M	ALS/-

CONTROL ID	SAMPLE DATE	AGE	GENDER	MATCHED TO
11	05/04/2012	55	F	P TO 3
5	12/04/2012	56	M	P TO 4
6	19/04/2012	47	F	NBLD REL 13
12	27/04/2012	57	F	P TO 8
10	15/04/2012	63	M	
14	17/05/2012	62	F	P TO 7
15	12/06/2012	69	F	P TO 17

Table indicating patient and control IDs used for this study, as well as blood sample extraction date, age, gender, diagnosis and whether patient was treated with Riluzole. Controls that were matched to patients are also indicated (M = male, F = female, R = treated with Riluzole, P = partner, NBLD REL = non-blood relative). Samples from underlined patients and controls were used in PCR validation.

### RNA extraction and Globin depletion

RNA was extracted by PAX (Qiagen), TEM (Applied Biosystems) and LL (Ambion) methods as described in the respective maufacturer's instructions, in each case omitting any DNase step. All samples were extracted separately and not pooled. The quality and yield of extracted RNA was analysed by examining electropherogram traces generated by the 2100 Bioanalyzer (Agilent) running the samples on a total eukaryote RNA nano chip, and by UV spectrography using Nanodrop 1000 (Thermo Scientific). Samples were stored at −80°C until required for globin depletion (TEM) or amplification (LL). Before globin depletion was carried out on TEM and PAX samples, PAX samples underwent DNase (NEB) treatment (2 µg of RNA, 2U DNase I) for 10 mins at 37°C followed by addition of EDTA to a final concentration of 5 mM and subsequent heat inactivation at 75°C for 10 mins. Samples were run on the 2100 Bioanalyzer to measure quality, and subsequent globin depletion (GlobinClear, Ambion) was carried out with 1 µg of RNA as described in the manufacturer's instructions. Where the RNA concentration was <67 ng/µl, samples had to be concentrated using 50 µg/ml Glycoblue (Ambion), in 0.5 M ammonium acetate, 50% isopropanol.

### RNA Amplification and Affymetrix microarray analysis

Linear amplification was employed using the Affymetrix GeneChip 3′ IVT Express Kit according to the manufacturer's instructions. 200 ng of total RNA was reversed transcribed to synthesize first-strand cDNA with oligo(dT) primers containing a T7 flanking sequence. After second strand synthesis, labelled aRNA was synthesized in an *in vitro* transcription reaction. In order to minimise batch effects, 10 samples were randomly chosen for each round of IVT. A number of samples had to be concentrated using 50 µg/ml Glycoblue (Ambion), in 0.5 M ammonium acetate, 50% isopropanol before fragmentation. 15 µg of aRNA was fragmented as described in the manufacturer's instructions and hybridized overnight onto U133 Plus 2.0 human genome arrays as previously described [24]. 12 arrays were hybridised per run and samples to be run were chosen in a similar random manner to the amplifications in order to minimise batch effects. After washing the chips in the Affymetrix Fluidics System 450, they were scanned in the GeneChip 30007G Scanner.

Quality control was initially carried out on Affymetrix expression console, on MAS5.0 normalised data. The data were also normalised by GCRMA for further quality control, gender differences using the program QLUCORE OMICS Explorer and analysis of globin isoform levels in GENESPRING (Agilent). CEL file data were analysed by the R package PUMA for compiling a list of differentially regulated genes. We utilised PPLR (probability of positive log-ratio), a tool within the PUMA suite that measures the uncertainty in estimation of gene expression, i.e. the technical variability, in order to provide a robust estimate of differential expression and listed significantly differentially regulated genes with a pLikeValue <0.05 (Liu et al., 2006). Gene Ontology and KEGG pathway analysis was carried out using the bioinformatics tool DAVID [26].

### Real-time PCR

200 ng of total RNA from 6 of the patients and 6 of the controls (**Table 5**) was reverse transcribed using the High Capacity RNA-to-cDNA Kit (Applied Biosystems) in reaction volume of 20 µl. This represented our best quality samples. As calculated from efficiency plots for each gene, samples of cDNA were diluted 1∶10, and 2.5 µl (2.5 ng) were used per PCR reaction for all genes except EHBP1, where 4 µl (4 ng) was used with 5x Brilliant III Ultra fast SYBR Green QPCR Master Mix (Agilent). In all cases primer concentrations were optimised to 300 mM and reactions carried out in triplicate. Real time PCR was carried out as previously described using the MX3000P cycler and MxPro software for analysis of Ct values. ΔCt values for each sample were calculated by subtracting Ct values from genes of interest from corresponding Ct values of the housekeeping gene GAPDH, which exhibited consistent expression across all samples. Subsequent ΔΔCt (ΔCtpat-ΔCtcont) were calculated in order to assess fold changes using the following formula: fold change  =  2^−(ΔΔCt)^. Unpaired two-tailed t-tests were used to determine whether differences in gene expression levels between patients and controls were statistically significant.

## Supporting Information

Table S1 **Gene Ontology of genes unmasked by globin depletion (top 100 GO terms).**
(XLSX)

Table S2 **List of statistically significant (pLikeValue <0.05) differentially regulated genes after PUMA analysis of LL samples (3047; FC, fold change).**
(XLSX)

Table S3 **List of statistically significant (pLikeValue <0.05) differentially regulated genes after PUMA analysis of TEMPUS GC samples (3619; FC, fold change).**
(XLSX)

Table S4 **Control analysis: the number of differentially regulated genes for each of the 5 random pairs of groups after PUMA analysis and the number of genes expressed as a percentage of the original study.** The percentages were averaged and standard deviation was also calculated (STDEV). The distribution of ALS patients between groups is also detailed.
(XLSX)

Table S5 **KEGG pathway analysis of LL affected pathways.**
(XLSX)

Table S6 **KEGG pathway analysis of TEM GC affected pathways.**
(XLSX)
